# Cellular Specificity of the Blood–CSF Barrier for Albumin Transfer across the Choroid Plexus Epithelium

**DOI:** 10.1371/journal.pone.0106592

**Published:** 2014-09-11

**Authors:** Shane A. Liddelow, Katarzyna M. Dzięgielewska, Kjeld Møllgård, Sophie C. Whish, Natassya M. Noor, Benjamin J. Wheaton, Renate Gehwolf, Andrea Wagner, Andreas Traweger, Hannelore Bauer, Hans-Christian Bauer, Norman R. Saunders

**Affiliations:** 1 Department of Pharmacology & Therapeutics, University of Melbourne, Melbourne, Australia; 2 Department of Neurobiology, Stanford University, Stanford, California, United States of America; 3 Institute of Cellular and Molecular Medicine, University of Copenhagen, Copenhagen, Denmark; 4 Department of Organismic Biology, University of Salzburg, Salzburg, Austria; 5 Institute of Tendon and Bone Regeneration, Paracelsus Medical University, Salzburg, Austria; INSERM U1094, University of Limoges School of Medicine, France

## Abstract

To maintain the precise internal milieu of the mammalian central nervous system, well-controlled transfer of molecules from periphery into brain is required. Recently the soluble and cell-surface albumin-binding glycoprotein SPARC (secreted protein acidic and rich in cysteine) has been implicated in albumin transport into developing brain, however the exact mechanism remains unknown. We postulate that SPARC is a docking site for albumin, mediating its uptake and transfer by choroid plexus epithelial cells from blood into cerebrospinal fluid (CSF). We used *in vivo* physiological measurements of transfer of endogenous (mouse) and exogenous (human) albumins, *in situ* Proximity Ligation Assay (*in situ* PLA), and qRT-PCR experiments to examine the cellular mechanism mediating protein transfer across the blood–CSF interface. We report that at all developmental stages mouse albumin and SPARC gave positive signals with *in situ* PLAs in plasma, CSF and within individual plexus cells suggesting a possible molecular interaction. In contrast, *in situ* PLA experiments in brain sections from mice injected with human albumin showed positive signals for human albumin in the vascular compartment that were only rarely identifiable within choroid plexus cells and only at older ages. Concentrations of both endogenous mouse albumin and exogenous (intraperitoneally injected) human albumin were estimated in plasma and CSF and expressed as CSF/plasma concentration ratios. Human albumin was not transferred through the mouse blood–CSF barrier to the same extent as endogenous mouse albumin, confirming results from *in situ* PLA. During postnatal development *Sparc* gene expression was higher in early postnatal ages than in the adult and changed in response to altered levels of albumin in blood plasma in a differential and developmentally regulated manner. Here we propose a possible cellular route and mechanism by which albumin is transferred from blood into CSF across a sub-population of specialised choroid plexus epithelial cells.

## Introduction

The brain develops and is maintained in a tightly controlled internal environment, protected from fluctuations of constituents of blood plasma by a set of mechanisms referred to as the blood brain barriers [1]. These exchange interfaces are present between brain endothelial cells (the blood–brain barrier proper), choroid plexus epithelial cells (blood–cerebrospinal fluid [CSF] barrier), pial surface (pia–arachnoid barrier) and between neuroependymal cells lining the ventricular system (CSF–brain barrier – present only in early development; [1]. The morphological basis of these barriers is dependent on intercellular junctions that occlude paracellular transfer of lipid insoluble molecules from the very earliest stages of brain development [1], [2]. In spite of this physical barrier the concentration of protein in fetal CSF is well known to be much higher than in the adult in all animal species studied [3]–[12]. In addition it has been shown previously that the proteins present in the CSF are mostly derived from blood plasma [10], [11], [13]–[15] and enter the ventricular system by a developmentally regulated transfer across a population of specialised choroid plexus epithelial cells [16]. The soluble and cell surface albumin-binding glycoproteins SPARC (secreted protein acidic and rich in cysteine) and GYPA and B (Glycophorin A and B) have been postulated to act as docking sites for albumin, mediating its uptake and transport by choroid plexus epithelial cells from blood into CSF [16]. We have recently proposed a model in which binding of SPARC to albumin acts as a shuttle, enabling transfer of the protein from the basal (plasma) membrane of albumin-transferring plexus epithelial cells though the blood–CSF barrier and into the CSF of the brain ventricular system (see also [16], [17]).

In the present study we wanted to investigate in more detail the possible involvement of SPARC in albumin transfer. In particular we examined whether or not SPARC is localised with sufficient proximity to its putative ligand, albumin, to suggest a molecular interaction. We also wanted to know if there were alterations in this association during brain development and during conditions in which the levels of circulating albumin were altered by introducing an exogenous, human albumin. We employed an *in situ* Proximity Ligation Assay (*in situ* PLA; trade name: Duolink) to determine the possible binding of endogenous mouse albumin and SPARC. We used the same assay to determine the binding specificity – i.e. whether mouse SPARC could also bind exogenous human albumin. In combination with this approach, we used specific antibodies to determine if the protein injected intraperitoneally (on the plasma side of the blood–CSF barrier) could be detected in the CSF – verifying its transfer into the CNS. Quantitative qRT-PCR was employed to detect changes in transcript levels for SPARC in choroid plexus during development and following injection of exogenous albumin. We found that SPARC is able to interact with mouse albumin from very early in development. In addition, this putative receptor/trafficking molecule (SPARC) is well placed to bind exogenous human albumin in the plasma, and very occasionally in the choroid plexus epithelium but only at older ages. These results suggest an albumin–SPARC complex at the blood–CSF interface. We also confirm species specificity of albumin transfer and indicate a developmentally regulated molecular mechanism by which this protein is transferred from blood into the CSF.

## Methods

### Ethics statement

Experiments were conducted in accordance with the Australian code of practice for the care and use of animals for scientific purposes 7th Edition, published by the National Health and Medical Research Council. All animal research protocols were reviewed and approved by the University of Melbourne Faculty of Medicine, Dentistry and Health Sciences Animal Ethics Committee and registered under ID. Number 1112115.

### Injections of exogenous human albumin

Postnatal day 2 (P2) and P10 pups and adult mice were injected intraperitoneally (i.p.) with two different doses (50 and 250 µg/g body weight) of exogenous human serum albumin (Sigma, St Louis, MO, USA). Control animals received an injection of a similar volume of 0.9% (w/v) sterile sodium chloride. Animals were left for 16–24 hours to obtain steady state CSF/plasma concentration ratios [10], [16].

### Animal preparation and collection of fluid and tissue samples

At the end of the experiments animals were terminally anesthetized with inhaled isoflurane. CSF was sampled from the *cisterna magna* with a glass microcapillary (outer diameter, 30–50 µm) attached to PVC tubing using gentle suction [18]. CSF samples were examined under a microscope for blood contamination. This method of verification can detect as little as 0.2% contamination [19]. Blood was sampled via direct cardiac puncture with a heparinized 20 gauge needle, and centrifuged at 1500 g for 5 min and plasma retained. All samples were stored at −20°C until required. Individual samples were pooled from entire litters (a minimum of two litters, *n* = 10 animals per sample).

Choroid plexuses were dissected out from lateral ventricles under cold RNase-free phosphate-buffered saline (PBS, pH 7.3), and placed in fresh ice-cold RNase-free PBS. Plexuses from animals from several litters were pooled (*n* = 10 animals per sample) and spun down, excess PBS was removed, and the plexuses were snap-frozen in liquid nitrogen and stored at −80°C until required. In some experiments whole brains were collected for immunohistochemistry and *in situ* Proximity Ligation Assays (*in situ* PLA), also known as Duolink (see below). These animals were perfuse-fixed transcardially with cold 4% paraformaldehyde made in 0.1 M phosphate buffer. Perfusions were at 70% cardiac output for 10 minutes, with post-fixation overnight at room temperature in Bouin's solution (Sigma). Fixed brains were washed in 70% ethanol until clear and embedded in paraffin wax as described previously [11], [20]. Groups of 3–4 animals per age from at least two different litters were collected for histological analysis.

### Extraction of mRNA and quantitative PCR (qRT-PCR)

Total RNA was extracted from samples of pooled tissue (n = 10 animals per sample) using the RNeasy Plus Mini kit including QiaShredder according to manufacturer protocols (Qiagen, Valencia, CA, USA). Approximately 10 µg of total RNA was processed from each sample. The concentrations and purity of total RNA samples were determined with a Nanodrop SD-1000 spectrophotometer. Single-stranded cDNA was synthesized with the Applied Biosystems High Capacity RNA–cDNA conversion kit (Applied Biosystems, Carlsbad, CA, USA). The amounts of RNA used for cDNA conversion were standardized to allow appropriate sample comparisons. The qPCR reaction was performed with SYBR Green chemistry, and gene expression determined using the ΔΔCt method [21]. Primers for target genes were designed with the OligoPerfect Designer (Invitrogen, Carlsbad, CA, USA) and are as follows: *Sparc* F – gagggcctggatcttctttc, R – cacggtttcctcctccacta; Transferrin receptor was used as a housekeeping gene (*Tfrc* F – tcgcttatattgggcagacc, R - ccatgttttgaccaatgctg). PCR reactions were completed in a 7900HT Fast Real-Time PCR System running SDS 2.4 software (Applied Biosystems, Foster City, California, USA). A final reaction volume of 10 µL was composed of 5 µL of SYBR Green Master Mix (SA Biosciences, Valencia, CA, USA), 1 µL of forward and reverse primers (final concentration, 2 µM), 2 µL of 1∶10 diluted cDNA, and 1 µL of RNase-free water. Controls without template or reverse transcriptase were prepared and analysed simultaneously and these negative controls did not produce an amplified product. Amplification conditions were 50°C for 2 min, 95°C for 10 min, followed by 40 cycles of 95°C for 0.5 min and 60°C for 1 min. Gene expression was determined from the Ct value for each sample, expressed as fold change relative to adult control samples (unless otherwise stated), which were given a value of 1.

### Pre-absorption of anti-albumin antibodies

**Figure 1:**
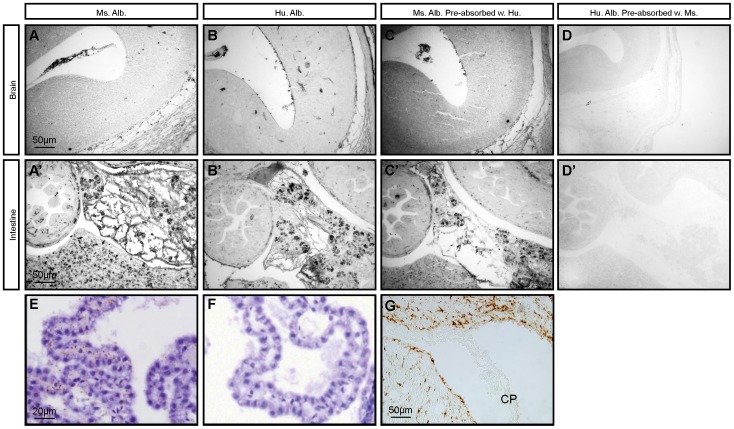
Pre-absorbtion of antibodies to mouse or human albumins. In order to remove cross-reactivity between antibodies to human albumin and endogenous mouse albumin, antibodies to mouse albumin were pre-absorbed with human albumin and antibodies to human albumin were pre-absorbed with mouse albumin prior to immunocytochemical staining. A/A′. A section through mouse brain (A) and abdominal cavity (A′) stained with antibodies to mouse albumin. Note strong, wide spread positive immunoreactivity in the intestine, while positive reactivity in the brain is only present in choroid plexus and blood vessels of the cortex. B/B′. Similar sections stained with antibodies to human albumin illustrating the level of cross-reactivity. C/C′. Section from the same animal immunostained with antibodies to mouse albumin pre-absorbed with human albumin. Note that the positive staining is reduced compared to un-absorbed antibody (A) but is still visible. However when anti-human albumin antibodies are pre-absorbed with mouse albumin (D/D′), all staining is removed (compare D with B). E&F. Results from *in situ* PLA assay of the adult choroid plexus using antibodies to human albumin pre-absorbed with mouse albumin on sections from human albumin injected mouse (E) and saline injected control animal (F). These figures illustrate that the pre-absorption protocol used (see Methods) was adequate to remove any cross-reactivity between the two albumins both at the immunocytochemical and *in situ* PLA levels. G. Additional control – GFAP immunoreactivity in P10 mouse brain and choroid plexus. Note that choroidal tissue is devoid of positive staining. Abbreviations: Alb., albumin; cp, choroid plexus; Hu., human; Ms., mouse. Scale bar is 50µm in A–D′, G; 20µm in E–F.

Commercially available antibodies to human albumin cross react strongly with endogenous mouse albumin (see Figure 1B/B′). To ensure we could properly detect human albumin distinct from mouse albumin we pre-absorbed antibodies to human albumin with purified mouse albumin and antibodies to mouse albumin with purified human albumin. This pre-absorption protocol removed all non-specific cross reactivity (Figure 1). Briefly, antibodies to mouse or human albumin were pre-absorbed with appropriate species-specific proteins in order to remove any possible cross-reactivity. The concentrations used were established empirically until possible cross-reactivity was abolished as checked by western blotting and immunocytochemistry. Briefly, 5µl of anti-mouse albumin antibody (abcam #ab19194, Australia) was pre-absorbed with 100µg of purified human albumin (Sigma) and 5µl of anti-human albumin antibody (DAKO, #A0001, Australia) was pre-absorbed with 100 µg of purified mouse albumin (Sigma #A3139). Samples were left at room temperature for 3–4 hours with gentle occasional mixing and transferred to 4°C for 16 hours. Samples were centrifuged for 10 minutes at 10000 g and supernatants removed. The supernatant was used as the primary antibody, when required, in the immunocytochemistry (Figure 1A–D), radial immunodiffusion and *in situ* PLA (Figure 1E–F).

### Validation of the absorption protocol

Immunocytochemistry: Five µm coronal paraffin-embedded brain sections were heated to 60°C and de-waxed in histolene, rehydrated in graded ethanol (100%, 95% and 70%, 5 minutes each), and incubated with protein and peroxidase blockers (DAKO) for 2 hours at room temperature before incubation overnight at 4°C with goat primary antibody against mouse albumin (abcam #ab19194, Australia, 1∶3000 dilution,), rabbit anti human albumin (DAKO, #A0001, diluted 1∶3000), pre-absorbed anti mouse albumin or pre-absorbed anti-human antibodies (as prepared above). This was followed by incubations with swine anti-rabbit immunoglobulin (DAKO, #Z0196, diluted 1∶200,) and rabbit Peroxidase anti-Peroxidase (1∶200, Sigma, #P1291) or anti goat immunoglobulin (DAKO, #Z0228, diluted 1∶200) and goat Peroxidase-anti Peroxidase (DAKO, #B0157, diluted 1∶200) at room temperature for 2 hours each. Sections were washed in PBS/Tween-20 buffer (3×5 minutes) between incubations. The reaction product was detected by exposure to DAB for 5 min, washed in distilled water, dehydrated in graded ethanol (70%, 95% and 100%, 5 minutes each), returned to histolene (2×5 minutes), and mounted with Ultramount #4 mounting medium (Fronine, Melbourne, Victoria, Australia). Positive staining was recognized as a brown colour. Negative control sections included those where the primary antibody was omitted; these always appeared blank. Additional controls included staining consecutive sections with rabbit anti GFAP (DAKO, #Z0334, diluted 1∶200) to confirm lack of non-specific staining in the choroid plexus (Figure 1G). Antibody dilutions were made in PBS/Tween-20 buffer with 0.2% fish gelatin (Sigma). The results of the pre-absorption are illustrated in Figure 1A–D′.

*In situ* PLA: The efficacy of removing possible cross-reactivity between anti-human albumin and mouse proteins (see below for details of the method) was tested on tissue from control (saline-injected) mice and resulted in no detectable signal (Figure 1E–F).

### Detection of SPARC and albumin by Western blot analysis

Mouse plasma samples were collected from embryonic, E15 and postnatal P2, P10, and adult animals. Equal amounts of total protein (as estimated by Bradford method [22] or BCA Protein Assay Kit, Thermo Scientific) were separated by SDS-PAGE on 10% polyacrylamide gels (BioRad, Hercules, CA). For detection of SPARC a total of 1µg per lane was loaded, whereas for albumin detection 0.5µg total protein was loaded. After blotting the resolved proteins onto PVDF membranes, unspecific sites were blocked and membranes were probed with the appropriate primary (anti-SPARC, Cell Signaling #5420, 1∶1000 and anti-mouse albumin, abcam #ab19194, 1∶1000) and HRP-labelled secondary antibodies (BioRad). Blots were developed using an enhanced chemiluminescence kit (BioRad) and signals were detected using a Chemidoc MP Imaging System (BioRad). Western blots were run on 3 separate samples collected and pooled from different litters and from separate adults.

### Radial Immunodiffusion

Radial immunodiffusion [23] was used to estimate concentration of human and mouse albumins in plasma and CSF samples collected from control (saline-injected) and experimental (human albumin injected) animals at P2, P10 and adult. When samples from control animals were used, antibodies to mouse albumin were applied without pre-absorption. For all other samples antibodies to human albumin were pre-absorbed with mouse albumin (Sigma) and antibodies to mouse albumin were pre-absorbed with human albumin (Sigma) as described above. Human albumin was used as standard for human albumin assay and mouse albumin was used as standard for mouse albumin assay. Plasma samples were diluted either 1∶100 (for human albumin) or 1∶500 (for mouse albumin) at all ages. For mouse albumin CSF was diluted 1∶20 at P2, 1∶10 at P10 and 1∶2 in the adult while for human albumin CSF samples were diluted 1∶5 at P2 and 1∶2 at P10 and adult. These dilutions were established experimentally to give the best range of precipitation rings [24]. Precipitation rings obtained after 48 hours of immunodiffusion in 1% agarose (Litex, UK) in Tris/barbitone buffer (pH 8.6) were measured and plotted against a standard curve constructed from serial dilutions of standard albumin concentrations [8], [24]. Three to six pairs of CSF and plasma samples were measured at all ages and obtained concentrations of albumin were used to calculate CSF/plasma concentration ratios [8], [14], [24].

### *In situ* Proximity Ligation Assay (*in situ* PLA, Duolink)

Tissue sections were de-paraffinized and rehydrated as described above and previously [16]. After endogenous peroxidase quenching and blocking (Roti-Immunoblock; Roth, Germany) for 1 hour at room temperature, tissue sections were incubated with respective primary antibodies (rabbit anti-SPARC, Chemicon/Millipore, AB1858, 1∶1000 and goat anti mouse albumin, abcam, ab19194, 1∶2500 or mouse anti human serum albumin [F-10], Santa Cruz, sc-271605; 1∶20) diluted in blocking reagent.

After overnight incubation at 4°C, sections were washed in TBS-Tween20 (2×5 minutes) and subsequently incubated for 1 hour at 37°C with the PLA probe-mix (anti-rabbit PLUS probe and anti-goat MINUS probe; 1∶5 dilution). The ligation and amplification reaction was performed according to the manufacturer's instructions. Amplification was terminated by washing the tissue sections three times for 5 minutes each in TBS-Tween20 at room temperature and peroxidase-labelled PLA probes were detected with NovaRED substrate and nuclear counterstaining was performed using the Duolink nuclear stain provided with the kit. Finally, tissue sections were dehydrated in a graded ethanol series (70–100%) and mounted in a non-aqueous mounting medium. Two specificity controls were performed using either a primary antibody targeting an unrelated epitope or by omitting the primary antibodies. In these cases the sections were always blank. Potential cross-reactivity between the antibodies used was removed and tested on sections from control (saline injected) animals (see above and Figure 1E–F).

### Photography and preparation of images

Digital photographs were taken with an Olympus DP70 camera attached to an Olympus BX50 microscope, and processed in Adobe Photoshop CS3. A×10 eyepiece and a×40 (0.65 NA) objective lens were used. The brightness and curve functions were used to obtain images with background close to white and to enhance contrast. No other manipulation of images was performed.

### Statistical analysis

One-way analysis of variance with Kruskal–Wallis *post hoc* test was performed with GraphPad Instat (version 3.06) software. A *p*-value <0.05 was considered a significant result. All data are expressed as means ± standard error of the mean unless otherwise stated.

## Results

It has been suggested previously that proteins present in the CSF are transferred across the blood–CSF barrier by a specialised cellular mechanism that is developmentally regulated [13], [14], [16], [20], [25]. Here we set out to describe the transfer of albumin into the CSF during mouse brain development by investigating the interaction of endogenous and injected exogenous (human) albumin with its putative transfer facilitator molecule: SPARC [16], [20].

### CSF/plasma concentration ratios for endogenous and exogenous albumins

**Figure 2:**
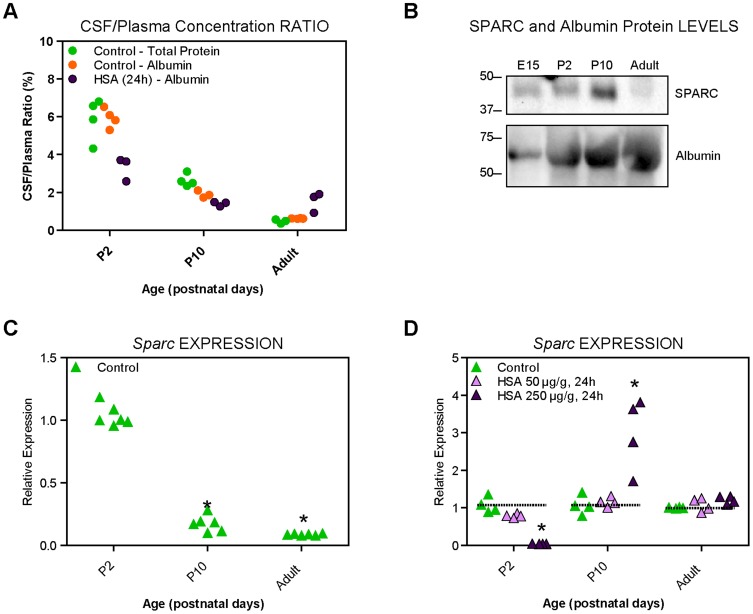
CSF/Plasma concentration ratios and *Sparc* expression in control and experimental mice. A. The method of Bradford (1976) was used to detect the total protein concentration of CSF and plasma samples taken from control animals, and those injected with human albumin for 24 hours. These data are represented as CSF/plasma concentration ratios (%). *, p<0.05, compared to age-matched control. Abbreviation: HSA, human serum albumin. Actual protein concentrations are presented in Table 1. B. Western blot analysis of SPARC and albumin in plasma of control mice during development. This analysis was performed 3 times on separately pooled samples. For SPARC 1 µg total protein was loaded in each lane; for albumin 0.5 µg total protein was loaded in each lane. Note that SPARC levels increase from E15 until P10 and decline in adults. C. Relative expression of *Sparc* in the lateral ventricular choroid plexus of control mice. Expression at P2 was taken as 1. *n* = 4 pooled biological samples (with 4 technical replicates each) in each instance. * *p*<0.05, one-way ANOVA compared to P2. Note developmental decline in the expression levels. D. Relative expression of *Sparc* in the lateral ventricular choroid plexus 24 hours following i.p. injections of human serum albumin. Animals were either injected (i.p.) with 0.9% w/v NaCl sterile saline (control) or human albumin at 50 µg/g or 250 µg/g. Relative expression refers to the expression of the gene in relation to age-matched controls. *n* = 4 pooled biological samples (with 4 technical replicates each) in each instance. * *p*<0.05, One-way analysis of variance with Kruskal–Wallis *post hoc* test, compared to age-matched control. Note a significant decline at P2 and an increase at P10 but only after the higher dose of exogenous albumin.

It is well known that the level of protein in the circulating plasma increases during development while simultaneously decreasing in the CSF [1], [7], [26] leading to a decrease of CSF/plasma concentration ratios – an index traditionally used as a measurement of penetration into the CSF ([26], [27] and see Discussion). Actual concentrations of total protein, mouse albumin and injected human albumin are presented in Table 1. Concentration ratios for total protein, mouse endogenous albumin and intraperitoneally injected exogenous human albumin, in P2 and P10 pups and in adults. are illustrated in Figure 2A and Table 1. We found that the CSF/plasma concentration ratio for total protein in postnatal mice decreased from around 6% at P2, to just over 2% at P10 and to just under 1% in the adult. (Figure 2A). Concentration ratios for endogenous mouse albumin were similar to those for total protein and were around 6% at P2, just under 2% at P10 and around 0.5% in the adult. In pups injected with 250 µg/g body weight human serum albumin, concentration ratios for injected exogenous human albumin reached levels that were lower by about 50% compared to mouse serum albumin in P2 (just over 3%) but were not significantly different at P10 (∼1.7%,) while in the adults in human albumin injected animals CSF/plasma concentration ratios were higher, (Figure 2A). The lower CSF/plasma concentration ratio of human albumin compared to endogenous mouse albumin at the earlier age confirms that during development choroid plexus is able to distinguish between different species of albumin and that this recognition mechanism is no longer present at later stages and in the adult [10], [13], [14], [25], where the apparent higher ratio for human albumin is most likely to be due to its clearance from plasma being faster than that from the CSF as has been described previously in other species [18], [19], [24]. No results for human albumin could be obtained for pups injected with the low dose of 50 µg/g body weight of protein as its levels, although detectable in blood plasma, were below detection limits of the method in the CSF (data not shown).

**Table 1:** Concentrations of total protein and albumins in CSF and Plasma of control and human albumin-injected postnatal mice.

Age	Plasma	CSF	Ratio
**Control – Total Protein**			
P2	3312.5±51.5	201.3±6.9	6.1±0.2
P10	4125.0±184.3 *	99.0±11.5 *	2.4±0.2 *
Adult	6187.5±51.5 *	27.7±0.6 *	0.4±0.01 *
**Control – Mouse Albumin**			
P2	2160.0±43.2	128.0±4.7	5.9±0.3
P10	2606.7±186.7	49.7±5.8 *	1.9±0.1 *
Adult	2757.5±122.5	17.1±0.7 *	0.6±0.01 *
**HSA 250µg/g (24 hours) – Human Albumin**			
P2	281.3±2.4	9.3±1.1	3.3±0.4
P10	146.0±2.2 *	2.0±0.1 *	1.4±0.1 *
Adult	184.7±6.4 *	2.4±0.1	1.2±0.03

Abbreviations: CSF, cerebrospinal fluid; HSA, human serum albumin; P, postnatal day. * *p*<0.05, One-way analysis of variance with Kruskal–Wallis *post hoc* test, compared to previous age in control animals. See also Figure 2.

### Western blot analysis of SPARC and albumin

**Figure 3:**
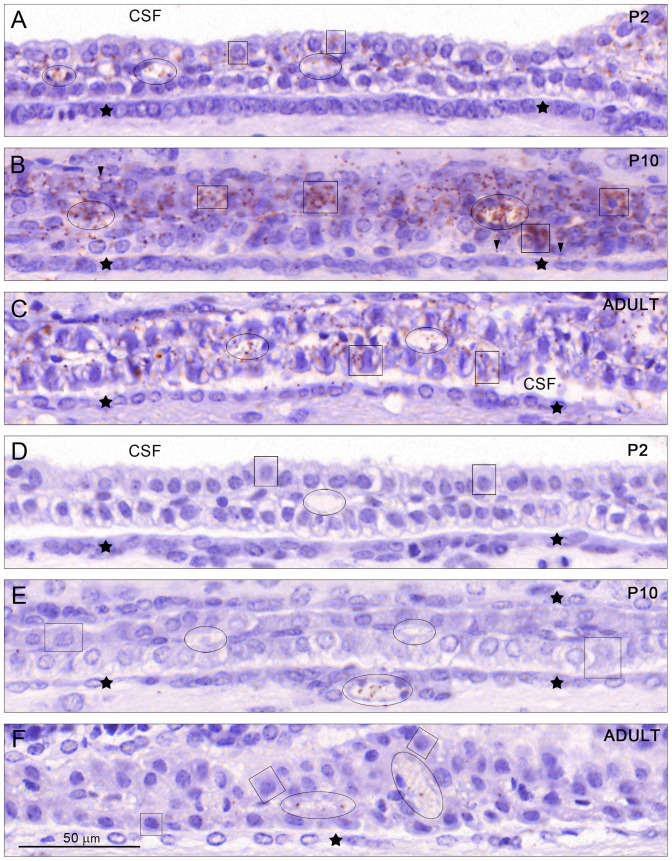
Overall tissue distribution of mouse albumin/SPARC. The distribution is illustrated using the *in situ* Proximity Ligation Assay (PLA); signals (red/brown dots) indicate close molecular proximity of albumin and SPARC in the lateral ventricular choroid plexus at P2 (A), P10 (B) and adult (C) and of human serum albumin/SPARC *in situ* PLA signals at P2 (D), P10 (E) and adult (F). In A note that some plexus epithelial cells outlined by boxes and plasma in blood vessels encircled by ovals show a discrete signal. B shows very strong staining at P10 both in epithelial cells, some of which are outlined by boxes and plasma in blood vessels, some surrounded by ovals. Some positive signals in the CSF were also detected (arrowheads in 3B). C is from an adult plexus where it was still possible to detect positive signals in many plexus cells, some of which are outlined by boxes and in blood vessels (outlined by ovals) at a higher intensity than at P2 but definitely at a lower intensity than at P10. In D (P2), E (P10) and F (adult) human serum albumin/SPARC *in situ* PLA signals were in general absent from the CSF and plexus epithelial cells (boxes), but were present in very low amounts in ependymal blood vessels (marked with oval) at P10 and in plexus blood vessels to some extent (ovals) in adult animals. CSF, cerebrospinal fluid. Asterisks indicate the ependymal lining of the lateral ventricle. A–F, same magnification, scale bar is 50 µm.

**Figure 4:**
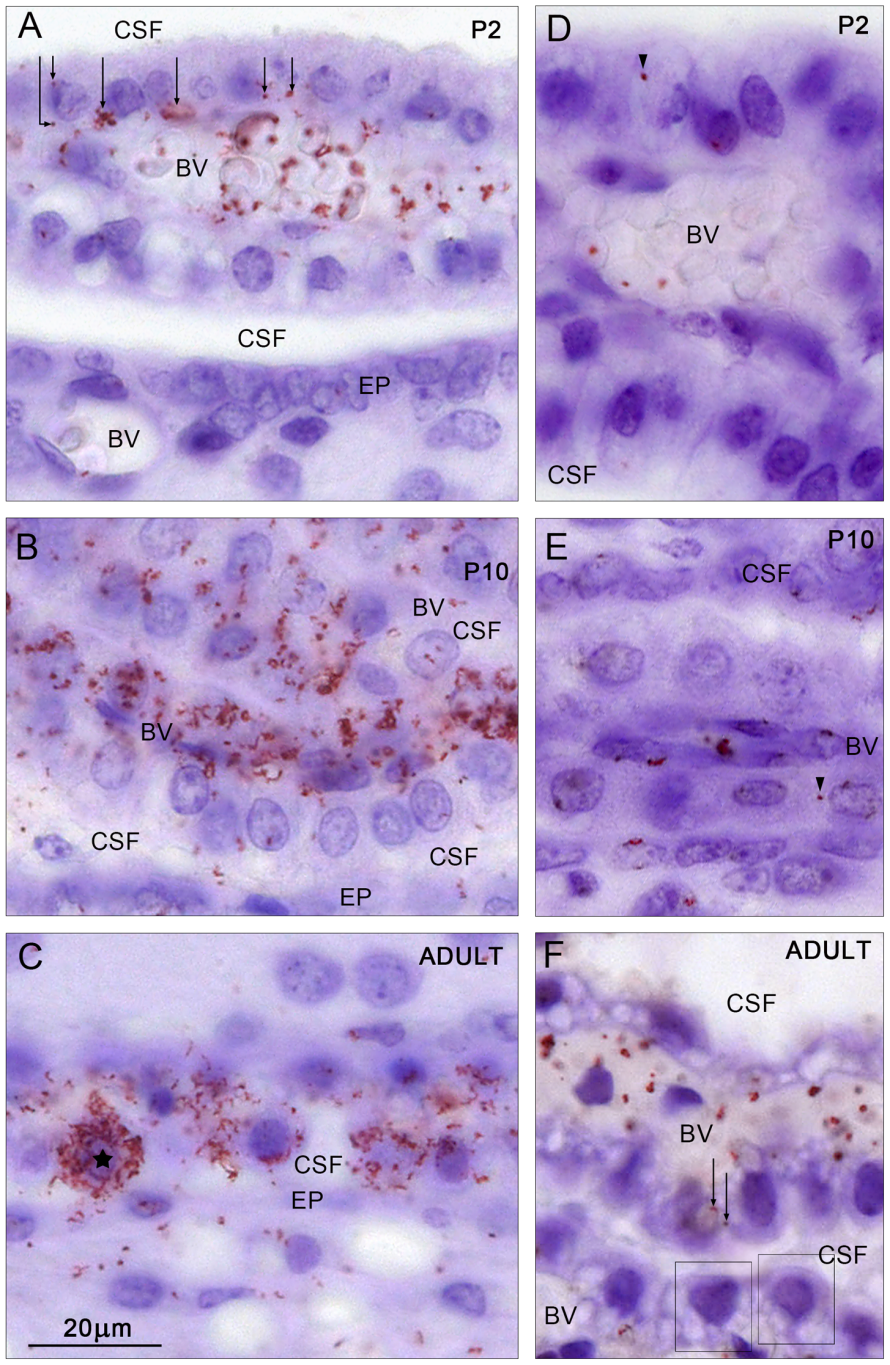
Cellular distribution of mouse albumin/SPARC. (A–C) and human albumin/SPARC (D–F) *in situ* PLA signals in the lateral ventricular choroid plexus at P2 (A,D), P10 (B,E) and adult (C,F). Note at P2 that most of the signal was distributed within blood vessels (BV), often associated with red blood cells. Under this magnification it is possible to distinguish positive signals distributed in the basolateral cytoplasm of choroid plexus epithelial cells (arrows). In contrast to the mouse albumin/SPARC signal, the human albumin/SPARC signal (D) was very rarely found and nearly always only associated with blood vessels (BV). Only one positive signal was found and it appears to be located in the extended extracellular space (arrowhead). At P10 (B and E) a very strong signal was visible for mouse albumin/SPARC (B) in many plexus cells distributed throughout the whole cytoplasm, blood vessels (BV) and also in the CSF. The human albumin/SPARC signal (E) was generally only present in blood vessels (BV) but a very occasional signal was detected in the apparent extended extracellular space (arrowhead). The CSF space was negative. In the adult (C and F) a mouse albumin/SPARC signal was distributed clearly throughout the cytoplasm of some choroid plexus epithelial cells (one cell marked with an asterisk). The positive signal was also detected in the ependymal (EP) and sub-ependymal layers of the brain. The human albumin/SPARC signal (F) was visible in blood vessels (BV) but not in the CSF and only very sporadically in the plexus epithelium (two positive red dots are indicated by arrows). Otherwise plexus epithelial cells (boxes) showed no *in situ* PLA signal. CSF, cerebrospinal fluid, EP, ependymal, BV, blood vessels. Same magnification, scale bar is 20 µm.

We have also analysed the amounts of both SPARC and albumin in serum from mice at E15, P2, 10 and adult. The amount of SPARC protein in the blood plasma, as detected by western blot analysis, increased from E15 to postnatal stages and was the highest at P10 followed by a marked decline in the adult (Figure 2B). In comparison the amounts of detected albumin steadily increased throughout the whole developmental period (Figure 2B). The results obtained for SPARC protein in plasma may explain the results illustrated in Figures 3 and 4 below that show the strongest signal for *in situ* PLA analysis at P10.

### Alterations in *Sparc* transcript expression

Changes in *Sparc* expression levels in the choroid plexus of P2, P10 and adult mice in control animals and following injection of human albumin are shown in Figure 2C–D. During development the results have been normalised to P2 values (expressed as 1, Figure 2C). Expression of *Sparc* in the choroid plexus was highest in the youngest brain (P2) and was much lower at P10 and in the adult. Following injections of human albumin the expression levels were affected in a developmentally regulated manner, but only after the higher dose of the protein (250µg/ml, Figure 2D). They were not significantly affected following a 50 µg/g i.p. dose of albumin (Figure 2D). In response to injections of human albumin, *Sparc* expression levels were reduced to almost zero at P2 while they were increased three-fold at P10 and were not different from control animals in the adult (Figure 2D). These data suggest that the responsiveness of *Sparc* gene to fluctuating levels of albumin in plasma is related to the stage of brain development that coincides with the timing when choroid plexus transfer mechanism changes from that able to discriminate between endogenous (mouse albumin) and exogenous (human albumin) at P2 to the one when both proteins are treated in the same way (P10 and adult).

### *In situ* PLA (Duolink) for mouse or human albumins and SPARC

**Figure 5:**
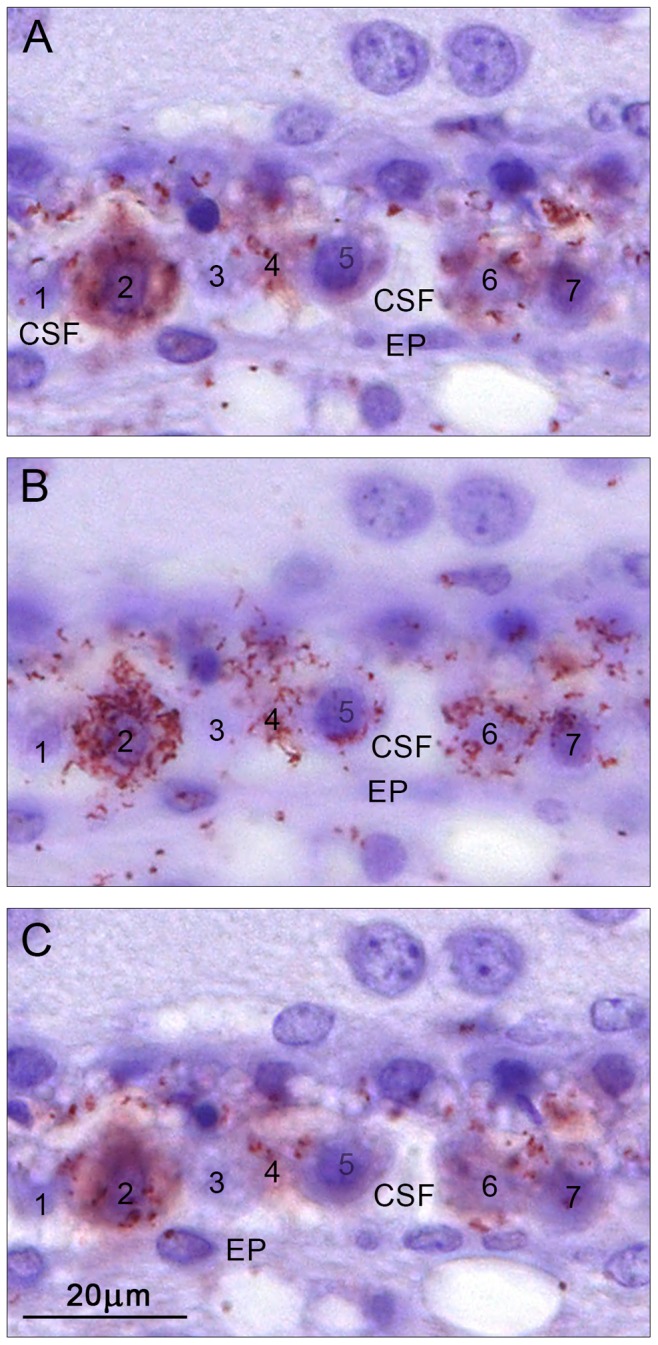
Subcellular distribution of mouse serum albumin/SPARC *in situ* PLA signals in the lateral ventricular choroid plexus epithelial cells in the adult. The same section is shown in three different planes of focus (A, B and C) to illustrate the 3-dimentional distribution of the signals within seven different epithelial cells (1–7). Note that no positive signal was visible in cells 1 and 3, cells 2 and 6 were completely filled by the reaction product while cells 4 and 5 showed some positive signals. Up to a certain density of *in situ* PLA signals, they appeared as discreet dots (cell 4 and 6 in B), whereas at higher densities the signals coalesce and appear as a diffuse label in the cytoplasm (cell 2 in A and C). In the optimal plane of focus (cell 2 in B) he signals seem to form a branching, continuous transcellular tubular system filled with the reaction product. CSF, cerebrospinal fluid; EP, ependyma; BV, blood vessel. A–C, same magnification, scale bar is 20 µm.

In order to establish whether albumin and its putative transport facilitator SPARC can be detected in close enough proximity to indicate their molecular interaction [28] *in situ* PLA was performed on paraffin embedded coronal sections of mouse lateral ventricular choroid plexuses at E15, P2, P10 and adult. Results are illustrated in Figure 3A–C as an overview of comparative regions of the plexus at different developmental ages to demonstrate tissue distribution of the *in situ* PLA signal. In Figure 4A–C the cellular distribution from the same brains is shown under high magnification while subcellular distribution of the signal in the adult choroid plexus epithelium is illustrated in Figure 5.

In addition we have used sections from brains of animals injected i.p. with human serum albumin to determine if we can detect differences in Duolink results between SPARC and endogenous mouse albumin or exogenous human albumin. This was conducted because of the results from *in vivo* permeability data showing that mouse choroid plexus transfer mechanism is able to distinguish between these two species of the protein (see below Figure 2A). Results are illustrated in Figures 3D–F and 4D–F.

### Mouse albumin and SPARC

At E15 and at P2 very few choroid plexus epithelial cells showed a clear positive signal for interaction of mouse albumin and SPARC (E15 not illustrated). Results for P2 are illustrated in Figure 3A and 4A. The signal was clearly visible within the cytoplasm of the plexus cells towards the basolateral membrane. Distinct positive signals could also be detected in blood vessels, often associated with red blood cells. Virtually no signal was found in the CSF space or in the ependymal cells lining the ventricular system (Figure 4A) in any of the sections studied.

P10 was characterised by the strongest and most widespread signal obtained (Figures 3B and 4B). At this age many choroid plexus cells showed intense positive signals including both basolateral and apical cytoplasmic compartments. In contrast to earlier stages positive signals were also detected in the CSF space and ependymal lining (Figure 4B). Stromal staining was also very prominent.

In the adult positive signals were distributed in a similar manner to that at P10 but at a lesser intensity (compare Figures 3B–C and 4B–C). An example of a strongly positive choroid plexus epithelial cell is indicated in Figure 4C. The subcellular distribution of the *in situ* PLA signal is illustrated in Figure 5. Apart from the cellular distribution of the signal in the plexus cells it was also clearly visible in the CSF, blood vessels, stroma and the ependymal lining (Figures 3C and 4C). These results indicate a change in the distribution of the *in situ* PLA signal between the earlier (E15 and P2) and later (P10 and adult) ages that coincides with the timing when the transfer across the blood–CSF barrier seems to change from the one able to distinguish between different species of albumin to when this protein specificity is no longer detectable (Figure 2).

### Human albumin and SPARC

At all ages the positive signal for albumin/SPARC interaction was always weaker for human serum albumin than for mouse serum albumin (compare Figure 3 panels A–C with D–F). This could be due to either a weaker binding between the two proteins or to a much lower concentration of exogenous injected human albumin (refer to Figure 2). In order to make sure that no signal was missed in our analysis and we were able to detect a positive reaction product, if present, sections at all ages were also developed with a higher concentration of the primary antibodies (Figure 4D–F), which resulted in over-stained material but allowed clear visualisation of *in situ* PLA signal in blood plasma, CSF and a very occasional choroid plexus epithelial cell but only at P10 and adult. No positive signal was ever observed at P2 at an intercellular localisation in the plexus. It was also noticeable that the number of positive signals in plasma increased with age and was the highest in the adult (Figure 4D–F).

### Subcellular distribution of mouse albumin/SPARC *in situ* PLA signal

Subcellular distribution of the positive signal from *in situ* PLA analysis for mouse albumin and SPARC in adult choroid plexus epithelial cells was investigated in more detail by observing consecutive planes of focus under high magnification in the light microscope and is illustrated in Figure 5. Similarly strongly stained cells were also observed at P10 but not at P2. Detailed analysis of the subcellular distribution of the positive signal in examples of these strongly stained cells in three focus planes of the cell suggests that the density of both mouse albumin and SPARC is so high that the signals coalesce giving an impression of a confluent pathway in which these molecules reside and are most likely transferred transcellularly through the cytoplasm from basolateral to apical membranes reminiscent of the tubulo-cisternal system described previously [29].

These results indicate that the molecular interaction between mouse albumin and SPARC is close enough to be detected by the *in situ* Proximity Ligation Assay particularly at older rather than younger ages. In contrast to the interactions between mouse albumin and SPARC, the interaction of human albumin and SPARC is virtually absent in the choroid plexus epithelial cells suggesting that the species specificity detected in the permeability experiments (see Figure 2) is conferred by the transfer mechanism present in the choroid plexus cells.

## Discussion

The aim of this study was to understand the cellular mechanism of albumin transfer across the blood–CSF barrier during brain development. We have proposed previously that several putative albumin-binding molecules could be involved in the process, based on immunocytochemistry and gene screening experiments [16], [17], [20]. In the present paper we present results indicating that endogenous albumin and SPARC are indeed present within individual choroid plexus cells in close enough proximity to indicate molecular interactions, especially at older ages and in the adult. In addition we show that the protein transfer system in the choroid plexus is species-specific and able to discriminate an exogenous, foreign albumin. This finding was confirmed by *in vivo* permeability data which showed that CSF/plasma concentration ratios in early postnatal animals for exogenous human albumin were lower than for endogenous mouse albumin, an observation well described for several other animal species [10], [13], [14], [25]. This species specificity is striking given the close sequence homology of different albumins. In the case of mouse and human albumin as studied here it is 72% and the homology for SPARC is 91%. Expression of this putative albumin transporter was also affected by increased concentration of albumin in blood plasma in a developmentally regulated manner.

A possible mechanism leading to endocytosis in the choroid plexus, i.e. which membrane receptor is responsible and if the process is indeed receptor mediated, has only recently been appreciated [16], [20]. It has been proposed that SPARC binds albumin and acts as a shuttle enabling transcytosis of albumin from the basal membrane of a subset of highly specific plexus epithelial cells, though the blood–CSF barrier, into the CSF of the brain ventricular system [16], [20]. Apart from the choroid plexus, in the central nervous system protein may also be taken up by early developing brain under certain pathological conditions of cellular stress such as inflammation [30]. In addition, albumin has been shown to be taken up by cells in the injured brain [31]–[33] and by growing tumour tissue, possibly as a source of amino acids and energy or as a by-product of uptake for albumin-bound metabolically important ligands [34].

### Relation between SPARC and albumin

Results presented in this study confirm that SPARC and albumin are indeed in close enough proximity to be interacting at the molecular level. It has also been demonstrated that human albumin is not transferred in the developing mouse across the blood–CSF barrier to the same level as endogenous mouse albumin, as has also been previously shown for other species [10], [13], [14], [25]. *In situ* PLA results illustrated clearly that the positive signal in the plexus epithelial cells (site of albumin transfer route; [11], [16], [20], [35]) was positive for SPARC and mouse albumin but very weakly or not at all positive for human albumin. However, the expression of *Sparc* transcript in the choroid plexus was affected by the increase of circulating human albumin in mouse plasma. This apparently contradictory result could be explained by the strong binding signal between human albumin and SPARC present in the plasma as detected by *in situ* PLA. This indicated that either the binding mode of albumin and SPARC in plasma is different from that within plexus cells or that SPARC is not the only transferring molecule involved in albumin trafficking in plexus epithelial cells. In addition, the results indicate that the mechanism of transfer that confers the species specificity resides in the epithelium of the plexus and not as a soluble form of SPARC circulating in blood plasma. Recent reports suggest that neonatal Fc receptor (FcRn), complexed with β2 microglobulin, is also involved in binding of albumin, acting as an important regulator of albumin distribution and half-life in blood [36]. In addition, binding of albumin to FcRn has been shown to be species-specific where human FcRn bound human and primate albumin avidly but not mouse or rat protein [36]. Our recent computer modelling (Kuiper *et al*., unpublished) indicates that the 3-D structure of FcRn and SPARC is remarkably similar providing a tantalising possibility that these molecules may form dynamic but changing complexes at the blood–CSF barrier allowing albumin transcellular transfer but also explaining the species-specificity observed in many previously described experiments in developing animals [10], [13], [25]. Gene transcripts for both FcRn and β2 microglobulin have been detected in the choroid plexus during development in the mouse [16] and the rat [17], [37]. Interestingly, levels of expression of FcRn receptor were reported to be higher at around P2–P9 than at earlier or later ages [17], [37], which seems to correlate with our results showing an increase in both the levels of SPARC in mouse plasma and *in situ* PLA signal at P10. Concentration of SPARC in mouse plasma was also the highest at P10 while its expression in the choroid plexus declined gradually from P2 until adulthood. Therefore it seems that there is a transition period around the first one to two weeks of life in rodents when albumin distribution in the body changes. The biological/developmental significance of this is not understood but may relate to some other physiological process during mouse development and is only a coincidental consequence in the choroid plexus. We have also shown previously that the proportion of albumin positive cells that express *Sparc* transcript is higher in the adult than during development [16]. In addition we have shown that the cellular distribution of SPARC protein, as shown by immunocytochemistry, is more widespread in the developing choroid plexus than that of albumin [16]. All these data together suggest that the relationship of SPARC and albumin is not linear and the mechanism of albumin binding and distribution in the circulation may well be independent of the mechanism operating in the choroidal epithelium. We propose that albumin transfer across blood–CSF interface is determined by a dynamic interplay of several possible transporters/facilitators that can change in development and possibly in response to changing physiological conditions. The results are, however, in agreement with the previously proposed hypothesis [10], [13], [14], [25] that early in brain development (in the mouse up to the first few days postnatal) the mechanism of albumin transfer across the plexus epithelium is able to recognise self from not self albumin and this stage is most likely to be involving more molecular interactions than just SPARC. On the other hand at older ages (in the mouse after P10) this mechanism is closely associated with SPARC but no longer discriminating between different species of the same protein. The subcellular distribution of albumin/SPARC *in situ* PLA signal is also compatible with the previously proposed transcellular route across the choroid plexus epithelium [29].

The expression of *Sparc* has been shown previously to be similar in all organ systems, including the brain, heart, kidney, liver, lung, thymus and spleen, with an average RNA-Seq log_2_FPKM expression value of 14.6 (range 11.6–17.2). There was a slight developmental decline in the expression of *Sparc* in heart, kidney, liver, lung and spleen in the second and third weeks postnatal in the rat and mouse; however, levels were constant from this time to adulthood [38], [39]. This moderate expression in whole organs is likely to be due to the quenching of highly expressing cell subtypes by lower expressing cells in the same tissue sample. In the brain *Sparc* expression is known to be high, in addition to the choroid plexus, in some cell types such as astrocytes in postnatal mice [40]. The binding of SPARC with albumin has mostly been studied in the context of peripheral capillary beds where albumin is known to bind to the endothelial glycocalyx. Sharing a likely albumin-binding domain with GP60, SPARC has been mostly overlooked as an albumin-binding molecule due to the fact that GP60 is the major mediator of albumin binding in peripheral microvascular endothelium [41]. SPARC has been suggested to play a role in angiogenesis in the developing mouse brain; *Sparc* mRNa was demonstrated by *in situ* hybridisation in pia mater, in the lining layer of the ventricles and in cerebral cortical blood vessels in the neonate [42]. Thus one possibility is that SPARC secreted into CSF and taken up across the neuroependyma may contribute to early brain vascularisation in addition to its proposed involvement in albumin transport into CSF.

In summary, the plasma protein albumin is a multifunctional carrier protein that has wide-ranging roles in the early developing CNS. From exerting homeostatic forces required for ventricular expansion [27] to the transport and distribution of a large number of blood-borne molecules (such as fatty acids, amino acids, hormones, metal ions, and many drugs, (e.g., [36], [43]). In addition, it has been suggested that albumin binding to barrier cell surfaces can create a more restrictive barrier as reported previously for endothelial cells [44], [45]. In this study, we demonstrated that following fluctuations in circulating levels of albumin, the expression of *Sparc* and the amount of SPARC protein change, in turn causing alterations in the amount of albumin entering the CSF. Like many other examples of albumin-binding transport mechanisms in peripheral systems (see [43]), choroid plexus SPARC-albumin binding is highly specific, with non-native albumin transferred to a lesser degree than endogenous mouse albumin. We have previously shown that SPARC is localised on the basolateral surface of the choroid plexus epithelium and is required for the facilitated delivery of native albumin and its bound ligands to the central nervous system [16]. Together with the data from the current study we suggest that this naturally occurring transport system could provide a potential novel entry method for the delivery into the CNS of drugs bound to albumin. Due to the lack of SPARC localisation on the internal surface of the plexus epithelium it could indicate a one-way and non-effluxable route that would maintain higher, more efficacious drug concentrations than previously available.
